# Activity of thonningianin A against *Candida albicans* in vitro and in vivo

**DOI:** 10.1007/s00253-023-12996-1

**Published:** 2024-01-11

**Authors:** Hui Wang, Hui Li, ZhiWei Liu, ZhenYu Zhu, YingYing Cao

**Affiliations:** 1 Shanghai Skin Disease Hospital, School of Medicine, Tongji University, Shanghai, 200443 China; 2 Department of Dermatology, Changhai Hospital, Naval Medical University, Shanghai, 200438 China; 3 School of Pharmacy, Naval Medical University, Shanghai, 200433 China; 4 Shanghai Engineering Research Center for Topical Chinese Medicine, Shanghai, 200443 China

**Keywords:** Thonningianin A, Antifungal, *Candida albicans*, ROS, CaMCA1

## Abstract

**Abstract:**

Fungal infections are increasing rapidly, and antifungal agents used in clinics are limited. Therefore, novel antifungal agents with high efficiency are urgently required. In this study, we investigated the antifungal activity of thonningianin A (THA), a natural compound that is widely found in plants. We first determined the activity of THA against *Candida albicans,* one of the most common fungal pathogens, and found that THA showed antifungal activity against all *C. albicans* tested, including several fluconazole-resistant isolates. THA also inhibits the growth of non-*Candida albicans* species. In addition, THA displayed antibiofilm activity and could not only inhibit biofilm formation but also destroy mature biofilms. The in vivo antifungal efficacy of THA was confirmed in a *Galleria mellonella* infection model. Further studies revealed that THA could enhance intracellular reactive oxygen species (ROS) production and regulate the transcription of several redox-related genes. Specifically, caspase activity and expression of CaMCA1, a caspase-encoding gene in *C. albicans*, were remarkably increased upon THA treatment. Consistent with this, in the presence of THA, the *Camca1* null mutant displayed higher survival rates and reduced caspase activity compared to the wild-type or CaMCA1-reintroduced strains, indicating an important role of CaMCA1 in the antifungal activity of THA. Taken together, our results indicate that THA possesses excellent antifungal activity and may be a promising novel antifungal candidate.

**Key points:**

• *THA exhibits activity against Candida species, including fluconazole-resistant isolates*

• *THA inhibits biofilm formation and destroys mature biofilm*

• *Elevated ROS production and CaMCA1-mediated caspase activity are involved in the antifungal mechanisms of THA*

## Introduction

**Fig. 1:**
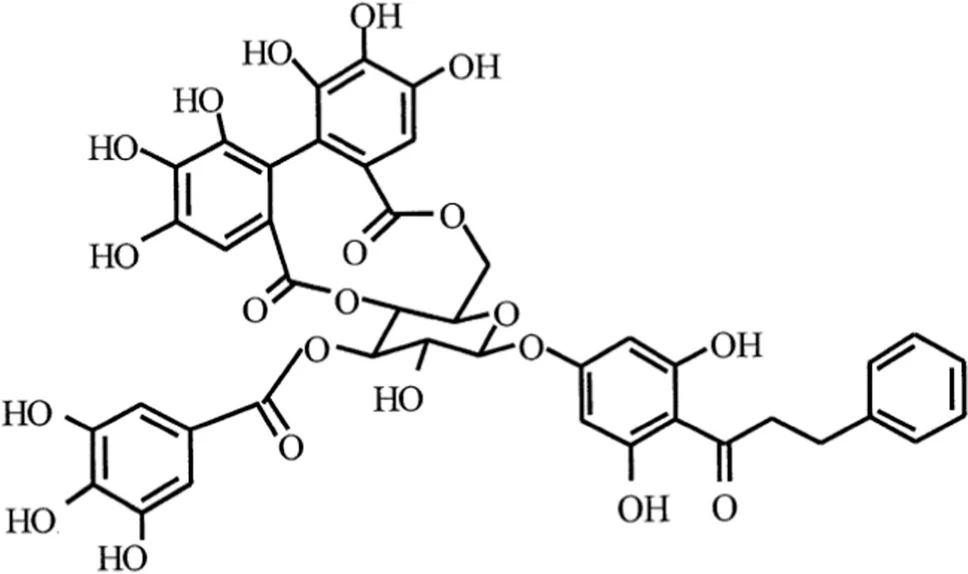
The structure of thonningianin A (THA)

Fungal infections have become increasingly common in recent years, especially in patients with immune systems weakened by cancer, infection with human immunodeficiency virus, or administration of immunosuppressive drugs (Pfaller and Castanheira 2016; Chang et al. 2017; Benedict et al. 2019). *Candida albicans* is one of the most common pathogens of fungal infection. When the human immune defenses become impaired, this fungus can be a common cause of superficial, mucosal, and systemic infections (Wang 2015). Unlike antibacterial drugs, clinical antifungal drugs are limited. Azoles (such as fluconazole), polyenes (such as amphotericin B), and echinocandins (such as caspofungin) are the three main types of drugs used in clinics against fungal species (Somer et al. 2011; Prasad et al. 2016; Wiederhold 2017). However, the disadvantages of these drugs often limit their wide clinical application. For example, amphotericin B can cause severe side effects such as fever, nausea, and vomiting (Laniado-Laborín and Cabrales-Vargas 2009). In addition, the antifungal spectrum of echinocandins is relatively narrow and the economic burden for the patients with echinocandins administration is much higher as compared to several other commonly used. Furthermore, the administration of azoles has resulted in drug resistance, and this is common with drugs such as fluconazole (FLC) antifungals. Due to its broad antifungal spectrum and low toxicities, fluconazole has a wide clinical application. However, long-term use of this drug inevitably leads to drug resistance, and fluconazole-resistant isolates are constantly emerging, which is a serious problem for antifungal therapy (Allen et al. 2015; Azevedo et al. 2015). Thus, there is an urgent need to develop novel antifungal agents, and the potential antifungal activities of plant-derived natural compounds are receiving increasing attention due to their extensive supply sources and easy accessibility. Many research groups have reported the activities of natural compounds against *C. albicans* and other fungal pathogens. For example, bacalein exhibited antifungal activity against *C. albicans* and non-*Candida* spp. (Kang et al. 2010; Lu et al. 2017; Aldholmi et al. 2019). Shikonin showed activity against both planktonic and biofilm growth of *C. albicans* (Yan et al. 2019). Other natural compounds that have been reported to exhibit antifungal activities include allicin, pterostilbene, roemerine, and so on (Khodavandi et al. 2010; Li et al. 2014; Ma et al. 2015). Thonningianin A (THA, Fig. 1) is an ellagic tannin flavonoid widely found in natural plants. Previous studies have revealed that THA possesses multiple biological activities, including antioxidant, antiproliferative, and anticancer activities (Gyamfi and Aniya 2002; Gyamfi et al. 2004; Lu et al. 2012; Huang et al. 2014; Zhang et al. 2015). Recent studies showed that THA could enhance microglial autophagy via the AMPK/ULK1 and Raf/MEK/ERK pathways; thus, this compound is effective in the therapy of Alzheimer’s disease (Huang et al. 2014; Zhou et al. 2022). In addition, THA was found to ameliorate vascular calcification in type 2 diabetes mellitus via the activation of L-type calcium ion channels (Shen et al. 2023). In this study, we aimed to investigate the antifungal activity of THA and explore its potential mechanisms of action.

## Materials and methods

### Strains, chemicals, and medium

The fungi used in this study included the *C. albicans* standard strain SC5314, CaMCA1-deleted (*Camca1△/Camca1△*), reintroduced (CaMCA1-EXP), and the corresponding wild-type strains (CAF2-1) (Cao et al. 2009). Other *Candida* species used in the experiments included the clinical fungal isolates of *C. albicans*, *C. parapsilosis*, *C. tropicalis*, *Nakaseomyces glabrata* (previous name *Candida glabrata*), and *Pichia kudriavzevii* (previous name *Candida krusei*). These strains were obtained from Shanghai Changhai Hospital and Shanghai Skin Disease Hospital (China) and were isolated from patients with invasive, mucosal, and superficial fungal infections. Fungal cells were routinely cultured in yeast peptone dextrose (YPD) medium. RPMI 1640 (Gibco, USA) was used to determine the minimum inhibitory concentrations (MICs) and antibiofilm activity of the drugs. FLC and THA were purchased from Macklin Biochemical Company (Shanghai, China) and Sigma-Aldrich Company (USA), respectively.

### Determination of minimum inhibitory concentrations

The MICs of THA and FLC were determined using the broth microdilution method as described previously with some modifications (Clinical & Laboratory Standards Institute 2017). The overnight-grown fungal cells were harvested and adjusted to 2 × 10^3^ cells/ml in RPMI 1640 medium. The concentrations of the drugs added to the plates ranged from 0.125 to 64 μg/ml. The plates were incubated at 35°C for 24 to 48 h. The optical density was measured by a microplate reader at 630 nm (OD_630_). The MIC_50_ and MIC_90_ values for the drugs were defined as 50 and 90% growth inhibition in the drug-treated groups as compared to the drug-free (control) group, respectively.

### Growth curve assay

The growth curve assay was performed as previously described (Quan et al. 2006). Briefly, *C. albicans* SC5314 or isolate 332 cells grown to exponential phase were diluted with RPMI 1640 medium to approximately 1 × 10^3^ cells/ml. The fungal cells were then exposed to different concentrations of THA or FLC and cultured at 30°C for 24 h with vigorous shaking (over 200 rpm). At each indicated time point, 10 μl of the culture was collected from each group, diluted, and incubated at 30°C for 48 h. The colonies were counted under a microscope and converted into log10 CFU/ml.

### Antibiofilm assay

The antibiofilm ability of THA was evaluated as previously described (Pierce et al. 2008). Briefly, the overnight-grown *C. albicans* SC5314 cells were adjusted to 1.0 × 10^6^ cells/ml in RPMI 1640 medium and added to 96-well tissue culture plates. Following the initial 90-min adhesion at 37°C the medium was aspirated, and fresh medium was added. The plates were further incubated at 37°C for 24 h. To monitor the effects of THA on biofilm formation, different concentrations of THA (ranging from 2 to 32 μg/ml) were added after 90 min of adhesion. To monitor the effect of THA on mature biofilms, the biofilms were allowed to form for 24 h, and fresh RPMI 1640 medium containing different concentrations of THA was added. The plates were then incubated at 37°C for an additional 24 h.

### XTT reduction assay

The antibiofilm activity of THA was measured using the XTT reduction assay, a reaction catalyzed by mitochondrial dehydrogenases (Ramage et al. 2001). Briefly, the grown biofilms were washed and treated with 0.5 mg/ml XTT and 1 mM menadione for 90 min at 37°C, then measured at 490 nm using a microtiter plate reader.

### Assessment of biofilm biomass

Biofilm biomass was assessed as previously described with some modifications (Nobile et al. 2006). The silicone disks (1.5 × 1.5 cm, Bentec Medical Corp., USA) used for determining the biofilm biomass were carefully weighed. To prepare for the biofilm biomass assay, disks were treated with bovine serum (Gibco, USA) at 37°C overnight and washed with PBS. After the pretreatment above, the disks were placed into 12-well tissue culture plates with one disk in each well. Next, the adjusted cell suspension (1.0 × 10^6^ cells/ml of overnight grown *C. albicans* SC5314 cells) was added to the plates. After 90 min of adhesion, a new RPMI 1640 medium containing THA was added to the well. The plate was further incubated at 37°C for 24 h with gentle agitation to allow for biofilm formation. The silicone disks were then collected and dried at room temperature to a constant weight. The biomass value was obtained by subtracting the mass of the empty silicone disk from that of the biofilm-grown silicone disk and further adjusting to obtain the silicone square weight.

### Scanning electron microscopy analysis

To observe the effect of THA on *C. albicans* biofilm formation with scanning electron microscopy (SEM), we developed *C. albicans* biofilms on silicon disks, as described above. THA was added to the wells containing the disks after 90 min of adhesion. The plate was further incubated at 37°C for 24 h with gentle agitation to allow for biofilm formation. The disks were then collected, washed three times with PBS, and fixed with 3% glutaraldehyde. After this, the disks were washed with 0.1 M Na_3_PO_4_ buffer (pH 7.2), dehydrated in ethanol, and coated with gold. The biofilms were observed by an environment SEM.

### *Galleria mellonella* assay

An in vivo killing assay using *Galleria mellonella* (*G. mellonella*) as the infection model was performed as previously described (Li et al. 2013). *C. albicans* SC5314 cells grown overnight were collected, washed, and adjusted to 1 × 10^8^ cells/ml. Each larva (approximately 300 mg) was then injected at the last left pro-leg with 5 μl of the cell suspension (containing 5 × 10^5^ cells). THA was delivered to the larvae 30 min after injection of the fungal cells. The death of the larvae was counted daily for 10 days. For the fungal burden assay, five larvae were homogenized 1 day after drug administration. The homogenate was incubated on YPD at 30°C for 2 days, and the log reduction in CFU/larva was calculated.

### Assessment of reactive oxygen species

Intracellular reactive oxygen species (ROS) production was assessed as previously described (Li et al. 2017). Overnight-grown *C. albicans* SC5314 cells were adjusted to 1 × 10^7^ cells/ml. Next, 20 μg/ml DCFH-DA (Molecular Probes, USA) was added, and the cells were cultured with constant shaking (200 rpm) at 30°C for 30 min. Subsequently, the cells were washed and treated with THA. At each indicated time point, the fluorescence values of the cells were determined using a multiple-mode microplate reader (Synergy H1, USA) with excitation at 485 nm and emission at 520 nm.

### Reverse transcription-quantitative PCR

The reverse transcription quantitative PCR (RT-qPCR) was performed as previously described (Yan et al. 2019). Briefly, overnight grown *C. albicans* SC5314 cells were washed and transferred to a fresh RPMI 1640 medium (1:100 dilution) and cultured with constant shaking (200 rpm) at 30°C for 4 h. The cells were then treated with THA (2, 4, or 8 μg/ml) and continually cultured under the same condition for 1, 2, or 4 h. For detecting the effect of THA on the gene expression in biofilms, overnight-grown *C. albicans* SC5314 cells were adjusted to 1.0 × 10^6^ cells/ml in RPMI 1640 medium and added to tissue culture plates. After initial 90 min of adhesion, the medium was aspirated, and a fresh medium containing 8 μg/ml THA was added. The plates were further incubated at 37°C for 24 h. Total RNA was extracted using a fungal RNAout kit (TIANZ, Beijing, China). Experiments were performed with the Light Cycler System (Roche Diagnostics, GmbH Mannheim, Germany). The PCR protocol consisted of an initial step at 95°C for 2 min, followed by 40 cycles of amplification and quantification (95°C for 10 s, 60°C for 20 s, 72°C for 15 s), and finally a cooling step to 40°C. The primer sequences for the genes detected are listed in Table 1. The mRNA levels were normalized to their 18S rRNA levels.

**Table 1:** Primers used in this study

Primer	Sequence
GRP2-F	TTCGTTTCTGGTGCTTCT
GRP2-R	TTTGTGGTCCATGAGTTT
CaMCA1-F	TATAATAGACCTTCTGGAC
CAMCA1-R	TTGGTGGACGAGAATAATG
SOD2-F	AACTTGGCTCCTGTCTC
SOD2-R	TATCACCATTGGCTTTG
SOD5-F	ACATTGGCGGTTTATC
SOD5-R	ATTACCTTGAGGAGCA
GLR1-F	GCTCATCTAAGTCATTGTGACC
GLR1-R	GCTGGACCAGAAGAAAAAGTTG
CAP1-F	ACCGTGAAGGTAAAGAACG
CAP1-R	GCTACCACCAGTATATTTAGCC
TRR1-F	TACGCCATTGGTCACATC
TRR1-R	CAAAGCAGCCATACATCC
EFG1-F	TATGCCCCAGCAAACAACTG
EFG1-R	TTGTTGTCCTGCTGTCTGTC
RAS1-F	GGCCATGAGAGAACAATATA
RAS1-R	GTCTTTCCATTTCTAAATCAC
ECE1-F	GCTGGTATCATTGCTGATAT
ECE1-R	TTCGATGGATTGTTGAACAC
HWP1-F	TGGTGCTATTACTATTCCGG
HWP1-R	CAATAATAGCAGCACCGAAG
ALS1-F	TTGGGTTGGTCCTTAGATGG
ALS1-R	ATGATTCAAAGCGTCGTTC
ALS3-F	CTAATGCTGCTACGTATAATT
ALS3-R	CCTGAAATTGACATGTAGCA
18S rRNA-F	AATTACCCAATCCCGACAC
18S rRNA-R	TGCAACAACTTTAATATACGC

Abbreviations: *F*, forward primer; *R*, reverse primer

### Caspase activity assay

For the determination of caspase activity, overnight-grown *C. albicans* SC5314 cells were washed and transferred to a fresh RPMI 1640 medium by 1:100 and cultured with constant shaking (200 rpm) at 30°C for 4 h. Subsequently, the cells were exposed to different concentrations of THA (4, 8, and 16 μg/ml) and continually cultured under the same condition for a certain time (3, 6, 12, and 24 h). Caspase activity was assayed by staining the *C. albicans* cells with D_2_R (CaspScreen flow cytometric apoptosis detection kit, BioVision), which could be cleaved into green fluorescent rhodamine 110 (Hao et al. 2013). Briefly, *C. albicans* cells treated with THA were washed and incubated with D_2_R at 30°C for 45 min before viewing and counting under a fluorescence microscope with excitation at 485 nm and emission at 520 nm.

### Statistical criteria

The results are presented as means and standard deviations. The data were analyzed using GraphPad Prism 6.0. A *P* value of < 0.05 or < 0.01 was considered statistically significant. The MIC data of the drugs were defined as 50 or 90% growth inhibition of the strains tested by comparing OD_630_ values of the drug-treated group with the drug-free (control) group.

## Results

### Antifungal activity of THA against *C. albicans*

**Fig. 2:**
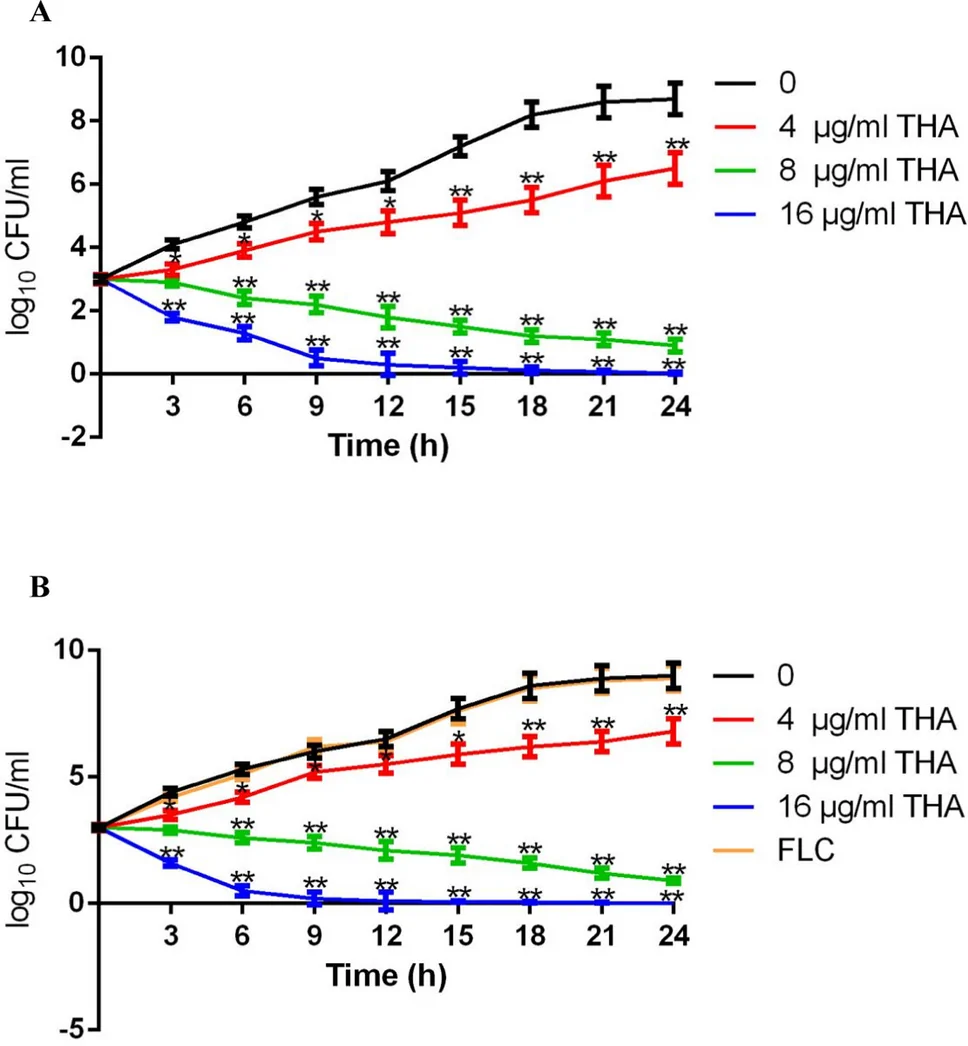
The growth curves were obtained by incubating *C. albicans* strain SC5314 (**A**) and fluconazole-resistant isolate 332 (**B**) with THA or/and fluconazole. The Log10 of CFU/ml remaining of the fungal cells upon exposure to 4, 8, and 16 μg/ml THA or/and 64 μg/ml fluconazole (FLC) were counted. Data are shown as the mean ± standard deviation of the independent assays in triplicate. *P < 0.05; **P < 0.01 as compared to the THA-free group

First, the activity of THA, which is an ellagic tannin flavonoid compound widely found in plants, against *C. albicans* was determined. The tested strains contained the widely used standard *C. albicans* strain SC5314 and several clinical isolates (including two fluconazole-sensitive and five fluconazole-resistant isolates obtained from patients with invasive, mucosal, or superficial fungal infections). The MIC_90_ value of THA against the *C. albicans* strains ranged from 2 to 8 μg/ml (Table 2). In the growth curve assay, THA at a dose of 8 μg/ml could remarkably inhibit the growth of SC5314 cells, with an approximately 1 × 10^4^ CFU/ml decrease being observed as compared to the control (drug-free) group at the time point of 12 h. Treatment with 16 μg/ml of THA resulted in a dramatic drop in the growth curve during the whole course of treatment (Fig. 2A). Moreover, THA possessed antifungal activity against fluconazole-resistant *C. albicans* isolates (such as isolates 589, 332, and 265) similar to that of fluconazole-sensitive isolates (such as isolates Y0109 and 638), with MIC_90_ values over 8- or 16-fold lower than that of fluconazole. For example, the MIC_90_ values of THA and fluconazole against the fluconazole-resistant *C. albicans* isolate 332 were 4 and > 64 μg/ml, respectively. The growth curve assay showed that treatment with 64 μg/ml fluconazole had no obvious growth inhibition on isolate 332, whereas the presence of THA exhibited dose-dependent antifungal activities. After treatment with 16 μg/ml THA for 12 h, the growth curve approached the *x*-axis, indicating strong antifungal activity (Fig. 2B).

**Table 2:** The MIC values of THA and fluconazole against *Candida* spp. after 24 and 48 h of incubation

Strains	Incubation period (h)	MIC_50_		MIC_90_	
		THA	FLC	THA	FLC
*Candida albicans*					
SC5314	24	1	0.125	4	0.25
	48	1	0.125	4	0.25
Y0109	24	1	0.125	4	0.25
	48	1	0.25	4	0.5
638	24	1	8	2	16
	48	2	8	4	16
647	24	0.5	16	4	32
	48	0.5	16	4	64
663	24	1	16	8	64
	48	1	32	8	64
589	24	2	> 64	4	>64
	48	4	> 64	8	>64
332	24	0.5	> 64	4	>64
	48	0.5	> 64	4	>64
265	24	1	32	8	>64
	48	1	> 64	8	>64
*Candida parapsilosis*					
561	24	1	1	4	2
	48	2	4	8	8
567	24	0.5	0.5	4	1
	48	1	1	4	2
582	24	2	0.5	8	2
	48	2	1	8	2
699	24	4	16	8	32
	48	4	32	8	64
*Candida tropicalis*					
467	24	2	2	4	4
	48	2	2	4	4
411	24	2	0.5	8	2
	48	2	2	8	4
489	24	1	2	4	4
	48	2	4	8	8
553	24	1	32	4	64
	48	1	32	4	64
*Nakaseomyces glabrata*					
773	24	1	16	4	64
	48	1	32	4	64
791	24	0.5	16	4	32
	48	1	16	4	32
741	24	1	32	2	64
	48	2	>64	4	>64
*Pichia kudriavzevii*					
213	24	1	32	4	64
	48	1	32	4	64
204	24	4	16	8	64
	48	4	64	8	>64
266	24	1	32	2	64
	48	2	64	4	>64

The unit of THA and fluconazole is micrograms per milliliter (μg/ml)

*MIC*_*50*_, MIC that inhibits 50% of the strain; *MIC*_*90*_, MIC that inhibits 90% of the strain

### Activity of THA against non-*albicans Candida* species

We next tested whether THA possessed antifungal activity against non*-albicans Candida* species. The MIC values of THA against *C. parapsilosis*, *C. tropicalis*, *Nakaseomyces glabrata*, and *Pichia kudriavzevii* were determined. As shown in Table 2, all non*-albicans Candida* species isolates tested were sensitive to THA treatment. The MIC_90_ values of THA against these isolates ranged from 2 to 8 μg/ml. It should be noted that, consistent with reports from other researchers (Beardsley et al. 2018), many non*-albicans Candida* isolates were less susceptible to fluconazole. Specifically, all *Nakaseomyces glabrata* and *Pichia kudriavzevii* isolates in this study were resistant to fluconazole. Thus, the similar MIC_90_ values of THA against *C. albicans* and non*-albicans Candida* species indicated a broad antifungal property of THA.

### Effect of THA on *C. albicans* biofilm

**Fig. 3:**
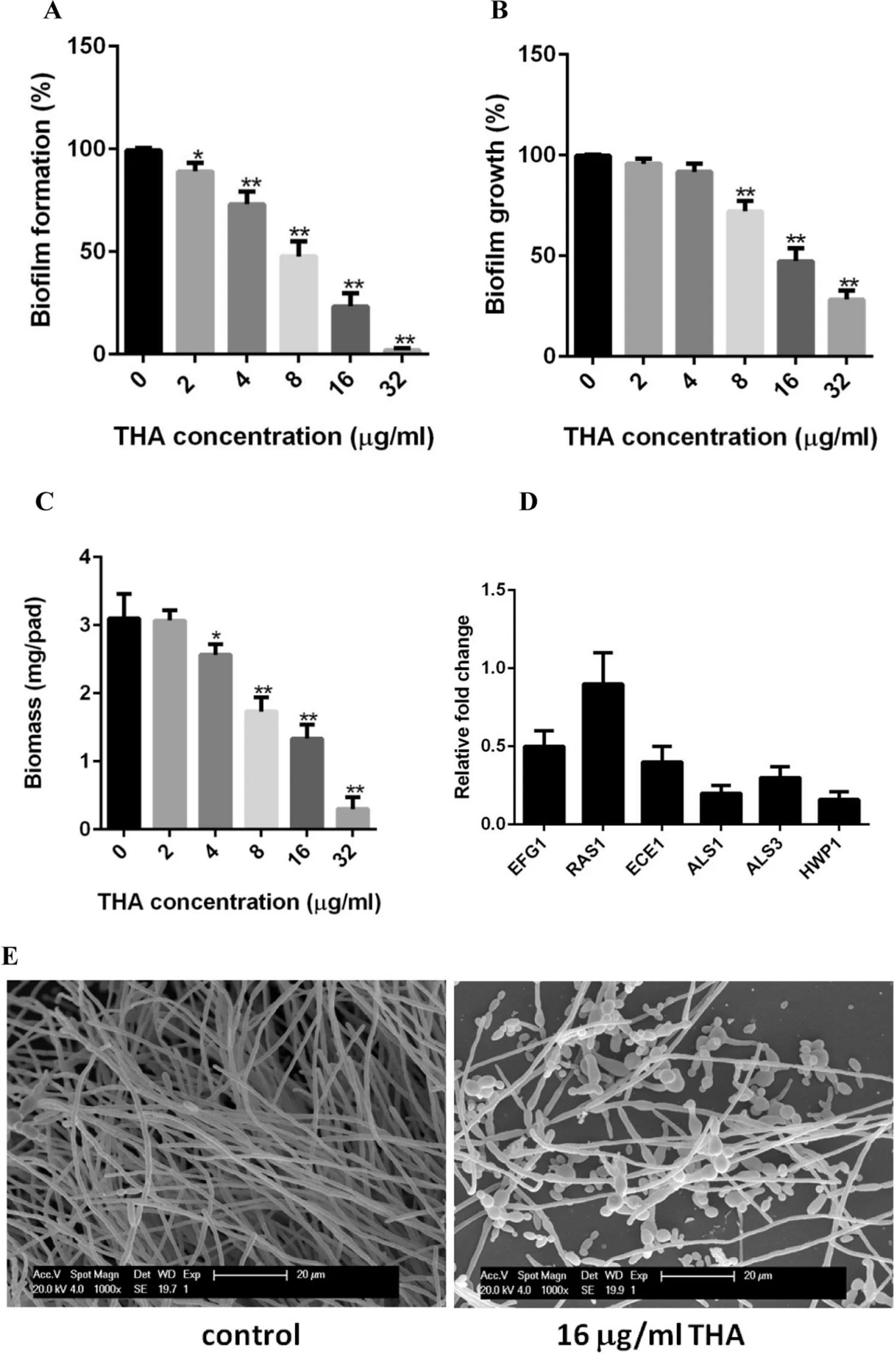
Antibiofilm activities of THA against *C. albicans* SC5314 biofilm detected by XTT reduction assay. **A** Effects of THA on biofilm formation. **B** Effects of THA on mature biofilms. **C** Effects of THA on the biomass production of the biofilms. The results were presented as the percent of THA-treated biofilms relative to the THA-free (control) biofilms. **D** RT-qPCR was used to detect the mRNA levels of the *C. albicans* genes when 8 μg/ml THA was used to inhibit biofilm formation for 24 h. The transcription of the genes was shown as the fold change in the THA-treated group relative to that of the THA-free group. **E** Effects of 16 μg/ml THA on biofilm formation, shown in SEM images. Data are shown as the mean ± standard deviation of the independent assays in triplicate. *P < 0.05; **P < 0.01 as compared to the THA-free group

The ability to form biofilms is an important virulence factor for *C. albicans*, often resulting in its high resistance to antifungal agents (Finkel and Mitchell 2011). Here, the antibiofilm activity of THA was determined. THA exhibited inhibitory activity on biofilm formation in a dose-dependent manner. The presence of 2 μg/ml THA displayed a weak but significant inhibition (*P* < 0.05) on biofilm formation, whereas 8 μg/ml THA showed a dramatic inhibition on biofilm formation. When 32 μg/ml THA was added, the biofilm formation was almost completely inhibited (Fig. 3A). Moreover, THA exhibited activity against mature biofilms. Upon exposure to 8 μg/ml THA, the growth of mature biofilm was significantly inhibited, and 32 μg/ml THA led to an even more severe impact on the mature biofilm (Fig. 3B).

To further confirm the antibiofilm effect of THA, we determined biofilm biomass upon exposure to THA. Consistent with the results obtained from the XTT reduction assay, THA could affect the production of biofilm biomass (Fig. 3C). Treatment with 4 μg/ml THA showed a remarkable influence on the production of biomass. The decrease in biomass production was even more obvious when the THA concentration was increased, and the addition of 32 μg/ml THA led to an approximately 10-fold drop in the biofilm biomass as compared to the drug-free biofilm. In addition, RT-qPCR revealed that THA could affect the expression of several genes involved in biofilm formation. As shown in Fig. 3D, when the biofilms were inhibited by 8 μg/ml THA for 24 h, the transcription levels of EFG1, ECE1, ALS1, ALS3, and HWP1 were remarkably downregulated, while the expression of RAS1 was almost not affected.

The effect of THA on biofilm was further detected with SEM. As shown in Fig. 3E, the biofilm formed by *C. albicans* SC5314 without THA treatment exhibited a three-dimensional structure and was mainly composed of long hyphae. The presence of 16 μg/ml THA seriously influenced the structure of the biofilm, which was composed of yeast cells, pseudohyphae, and less dense hyphae.

### Antifungal activity of THA in vivo

**Fig. 4:**
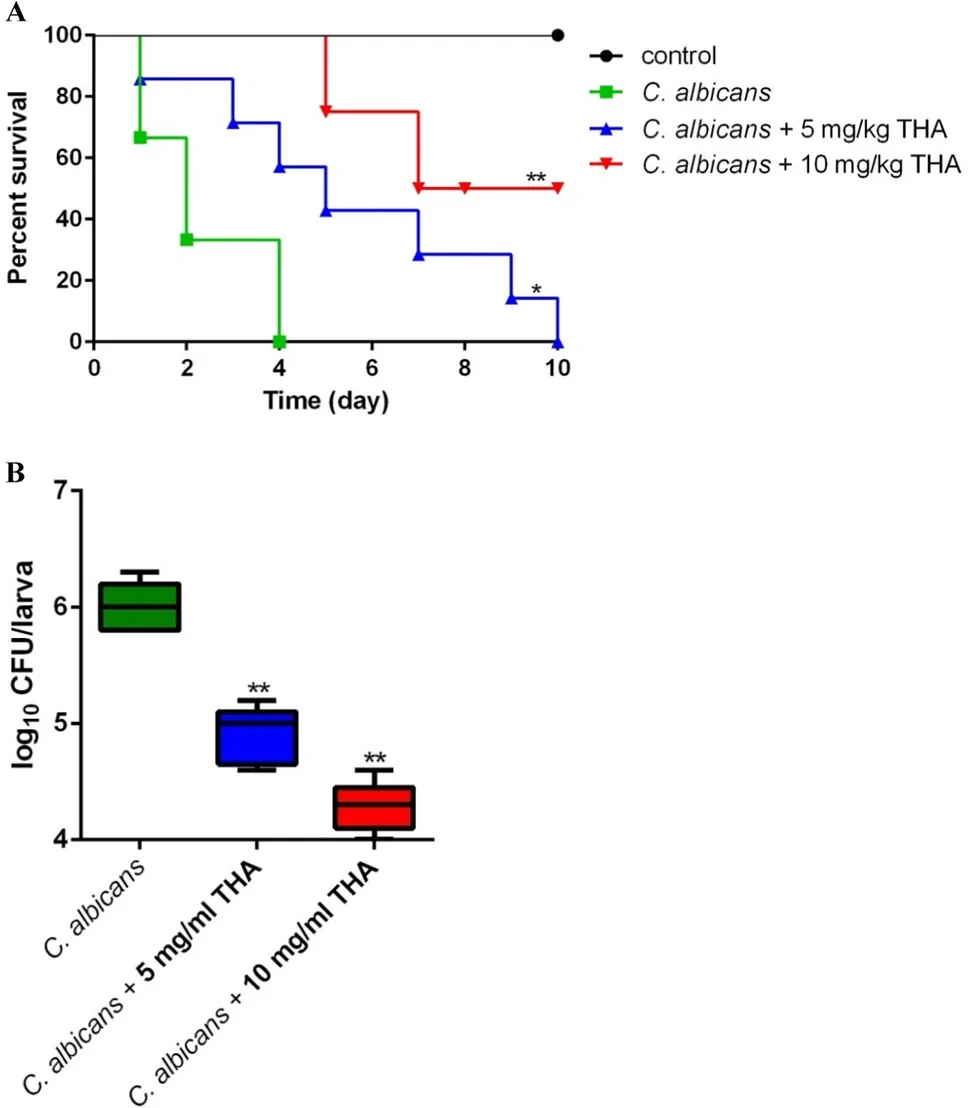
**A** The survival of *G. mellonella* larvae (*n* = 15) infected with *C. albicans* SC5314 cells and treated with THA (5 and 10 mg/kg) 30 min after infection. **B** The fungal burden of the larvae (*n* = 5) infected with *C. albicans* SC5314 cells and treated with THA (5 and 10 mg/kg) 1 day after infection. Statistical significance among the groups was analyzed by one-way ANOVA. *P < 0.05; **P < 0.01 as compared to the THA-free group

The *in vivo* activity of THA against *C. albicans* was determined using a *G. mellonella* infection model. All *G. mellonella* infected with *C. albicans* died within four days when no drug was administered (Fig. 4A). Administration of 5 mg/kg THA resulted in an obviously prolonged survival period with *G. mellonella* alive for 10 days after infection. When 10 mg/kg THA was administered, the mortality rate of *G. mellonella* remarkably dropped, with approximately 50% of *G. mellonella* living for more than 10 days.

We further investigated the *in vivo* activity of THA using fungal burden analysis. In the absence of THA treatment, a high fungal burden was observed in the *C. albicans*-infected *G. mellonella* (Fig. 4B). Administration of 5 mg/kg THA led to a significant decrease in fungal burden. Treatment with 10 mg/kg THA caused an even more obvious decrease in fungal burden, with an approximately 100-fold reduction as compared to the drug-free group.

### THA induces intracellular ROS production

**Fig. 5:**
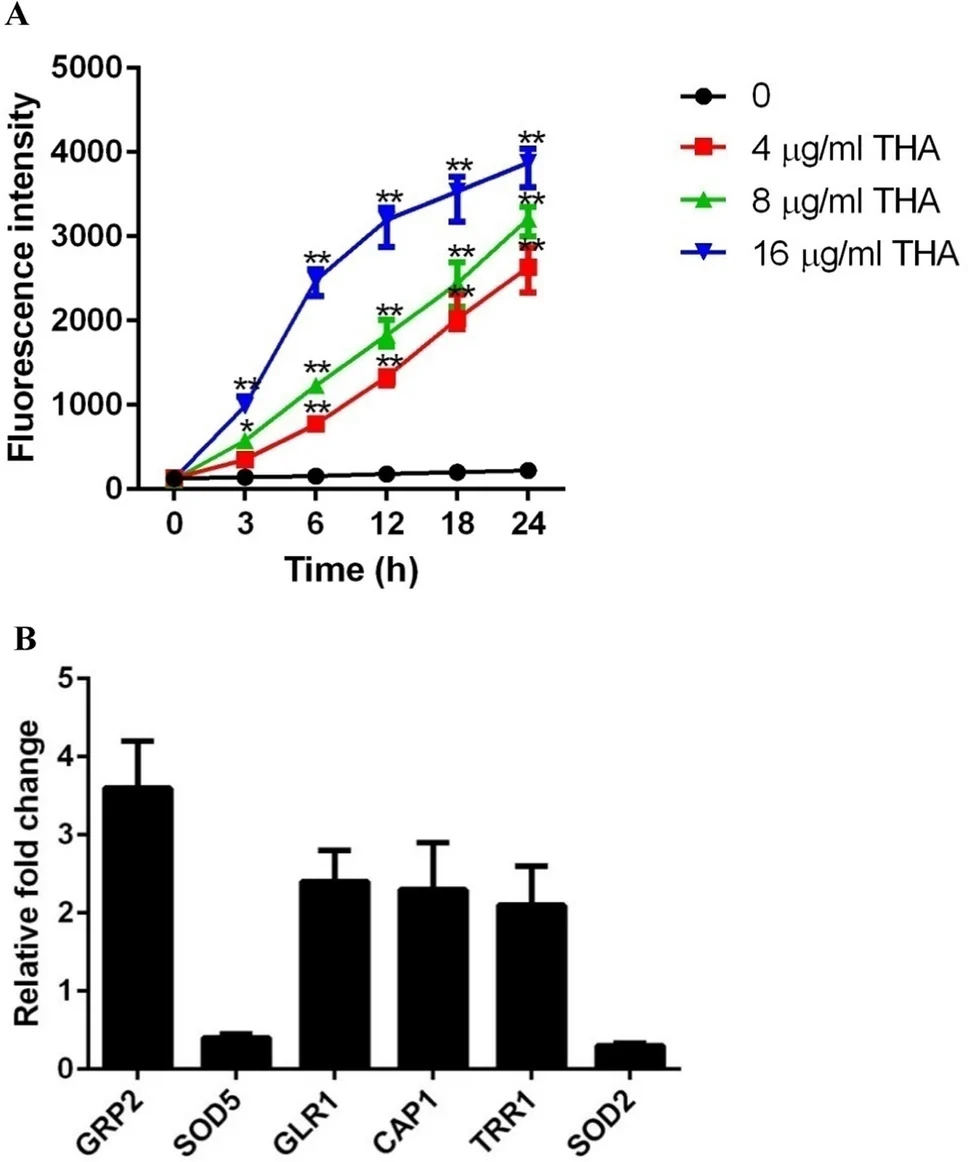
**A** THA increased intracellular ROS production. *C. albicans* SC5314 cells were exposed to 4, 8, and 16 μg/ml THA, respectively. **B** THA affected the transcription of the genes involved in oxidative stress. The *C. albicans* SC5314 cells were exposed to 4 μg/ml THA for 4 h. The transcription of the genes is shown as the fold change in the THA-treated group relative to that of the THA-free group. Data are shown as the mean ± standard deviation of the independent assays in triplicate. *P < 0.05; **P < 0.01 as compared to the THA-free group

Since oxidative damage caused by ROS is an important mechanism of action for antifungal agents (Ghannoum and Rice 1999; Kobayashi et al. 2002; Zhao et al. 2016), we tested the effect of THA on intracellular ROS production. A remarkable ROS elevation was observed in *C. albicans* cells upon exposure to THA (Fig. 5A). The addition of 4 μg/ml THA significantly stimulated the production of intracellular ROS. The presence of 16 μg/ml THA led to rapid and strong ROS production. At the time point of 6 h, the ROS levels were approximately three times higher in the 16 μg/ml THA group than in the 4-μg/ml THA group.

Next, we evaluated the mRNA levels of several redox-related genes in *C. albicans* using RT-qPCR. The addition of THA resulted in the upregulation of GRP2 (a NADPH-dependent methylglyoxal reductase, 3.6-fold increase), GLR1 (a glutathione reductase gene, 2.4-fold increase), CAP1 (a transcription factor specific to the oxidative stress response, 2.3-fold increase), and TRR1 (a thioredoxin reductase gene, 2.1-fold increase). However, the expression of two manganese superoxide dismutases, SOD2 and SOD5, was downregulated by approximately 3- and 2-fold, respectively (Fig. 5B).

### THA enhances caspase activity and CaMCA1 expression

**Fig. 6:**
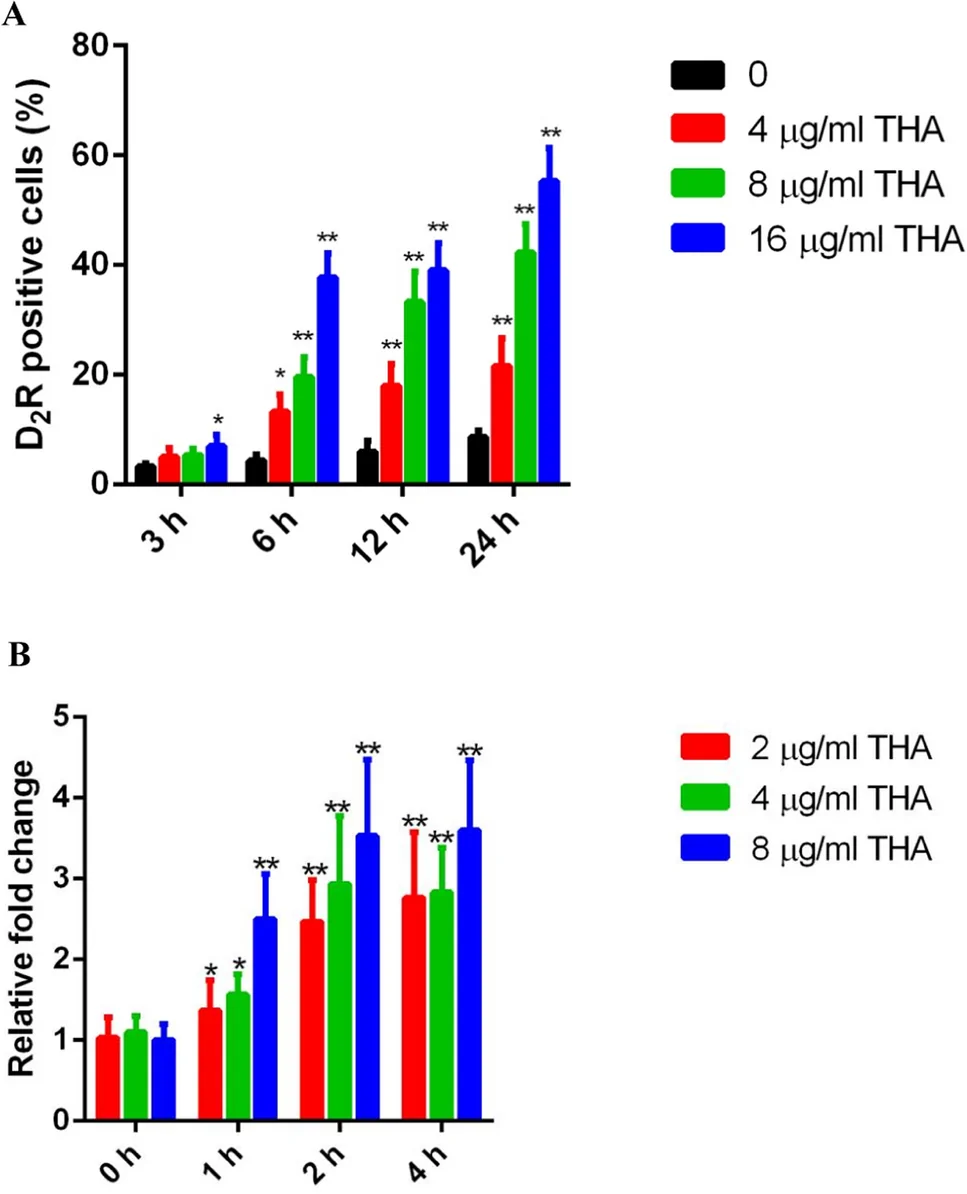
Effect of THA on the caspase activity and CaMCA1 expression. **A** *C. albicans* SC5314 cells were exposed to 4, 8, and 16 μg/ml THA, respectively. At the time points of 3, 6, 12, and 24 h, the caspase activity was detected. **B** THA affected the transcription of CaMCA1. The *C. albicans* SC5314 cells were exposed to 2, 4, and 8 μg/ml THA, respectively. The transcription of CaMCA1 was detected at the time points of 1, 2, and 4 h and shown as the fold change in the THA-treated group relative to that of the THA-free group. Data are shown as the mean ± standard deviation of the independent assays in triplicate. *P < 0.05; **P < 0.01 as compared to the time point of 0 h

In *C. albicans*, increased intracellular ROS levels are closely related to high caspase activity (Al-Dhaheri and Douglas 2010; Lu et al. 2011). Thus, the caspase activity in *C. albicans* cells was evaluated by staining the cells with the fluorescent dye D_2_R. As shown in Fig. 6A, the addition of THA resulted in a remarkable impact on caspase activity. At the time point of 24 h, 4 and 8 μg/ml THA increased caspase activity by 2.4- and 4.7-fold (*P* < 0.01), respectively. When the fungal cells were treated with 16 μg/ml THA for 24 h, the caspase activity increased by approximately 6 times compared to that in the control (drug-free) group.

The expression of CaMCA1, which encodes a metacaspase and is responsible for caspase activity in *C. albicans*, was analyzed using RT-qPCR. Consistent with the stimulation of caspase activity by THA, expression of CaMCA1 was enhanced upon THA treatment. Although 2 and 4 μg/ml THA showed a slight effect on the level of CaMCA1 mRNA at the time point of 1 h (1.37- and 1.57-fold increase, respectively), the presence of 8 μg/ml THA significantly upregulated the mRNA level of CaMCA1 at this time point (2.5-fold increase). When the fungal cells were exposed to 8 μg/ml THA for 4 h, the level of CaMCA1 mRNA increased by 3.6-fold compared to that in the control group (Fig. 6B).

### The Camca1 mutant shows resistance to THA

**Fig. 7:**
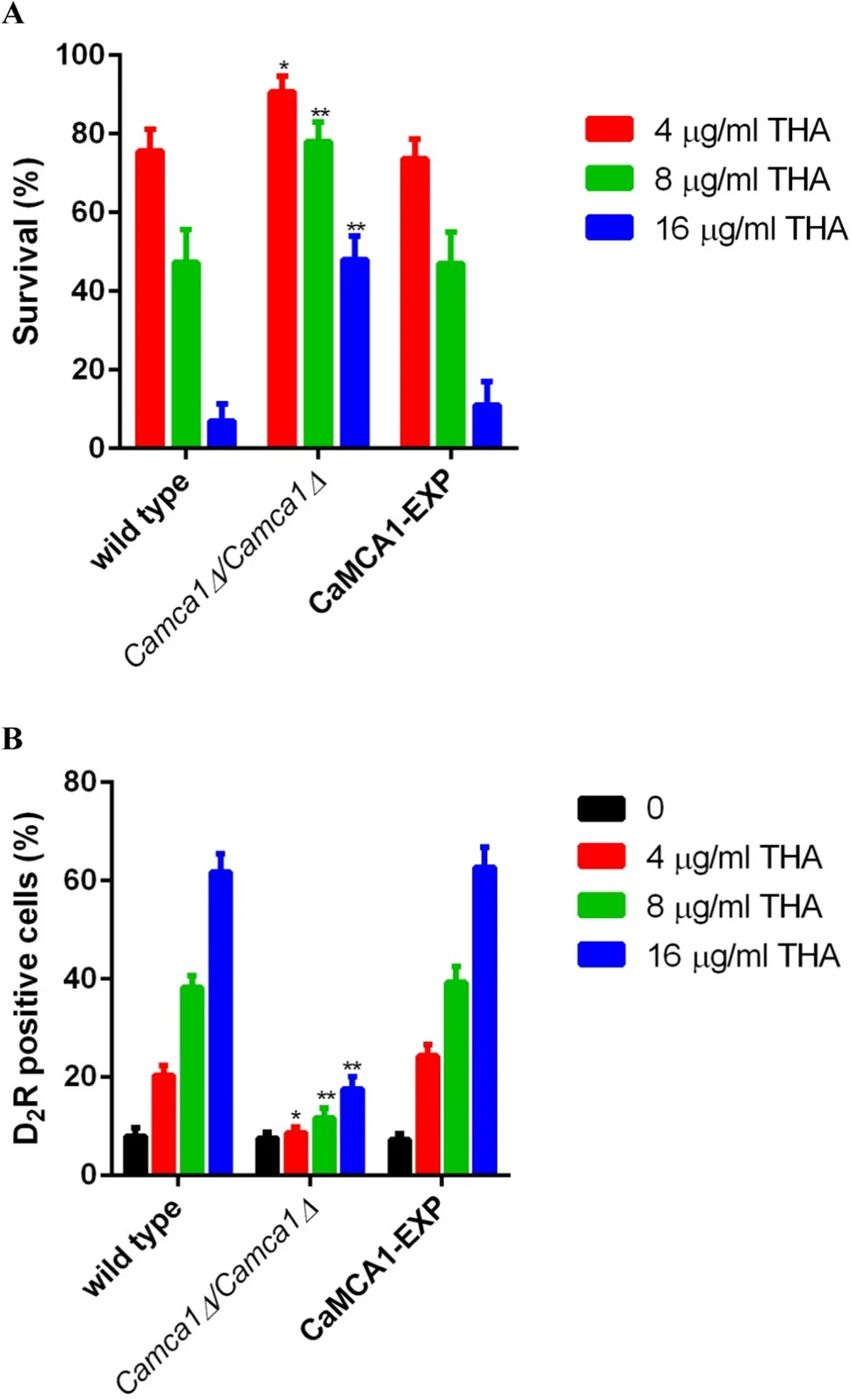
**A** Effect of CaMCA1 on THA-induced cell death. The *C. albicans Camca1* mutant (*Camca1△/Camca1△*), CaMCA1-reintroduced (CaMCA1-EXP), and wild-type (CAF2-1) strains were exposed to 4, 8, and 16 μg/ml THA for 12 h. The cells were then washed twice with PBS and plated on YPD agar plates. The fraction of viable cells was determined by counting the colonies and calculating the percentage of survived fungal cells relative to the control drug-free cells. **B** Effect of CaMCA1 on THA-induced caspase activity. The *C. albicans* strains were exposed to 4, 8, and 16 μg/ml THA for 12 h, and then the caspase activity was detected. Data are shown as the mean ± standard deviation of the independent assays in triplicate. *P < 0.05; **P< 0.01 compared with values from the control wild-type cells

In view of the enhanced transcription of CaMCA1 in *C. albicans* cells upon THA treatment, we evaluated the role of Camca1 in the antifungal effect of THA. As shown in Fig. 7A, there was an obvious difference in the survival rates between the *Camca1* mutant (*Camca1△/Camca1△*) and wild-type cells (CAF2-1) upon THA treatment. When 4 μg/ml THA was added, the survival rates of the wild-type, *Camca1* mutant, and CaMCA1-reintroduced (CaMCA1-EXP) strains were 75, 91, and 73%, respectively. Consistently, the *Camca1* mutant displayed lower caspase activity than the wild-type and CaMCA1-reintroduced cells (Fig. 7B). The effect of CaMCA1 deletion on the cell sensitivity to 16 μg/ml THA was even more obvious, as the survival rate of the *Camca1* mutant (48%) was approximately 7 times higher than that of the wild-type cells (7%). Consistent with this, the caspase activity in the wild-type, *Camca1* mutant, and CaMCA1-reintroduced strains was 66, 17, and 62%, respectively, in the 16-μg/ml THA-treated group.

## Discussion

Currently, fungal infections are common and have become a serious threat to immunocompromised patients (Jenks et al. 2020). Unlike a wide variety of antibacterial drugs, antifungal drugs are limited and often have disadvantages, such as unbearable adverse drug reactions, ineffectiveness against drug-resistant strains, and high cost, which often results in the premature termination of antifungal therapy. Our results showed that THA, a natural compound widely present in plants, possessed potent activities against *C. albicans*. Moreover, THA displayed inhibitory effects on *C. albicans* isolates resistant to fluconazole, which is one of the most commonly used antifungal agents, resulting in the rapid emergence of resistant isolates. The antifungal activities of THA were further confirmed by a growth curve assay, in which THA exhibited a dose- and time-dependent manner against both fluconazole-sensitive and fluconazole-resistant *C. albicans* strains. These results suggest that THA is effective against resistant *C. albicans* and may possess a mechanism of action different from that of fluconazole. In addition, THA showed activity against a series of non-*Candida* spp*.*, including *C. parapsilosis*, *C. tropicalis*, *Nakaseomyces glabrata*, and *Pichia kudriavzevii*, indicating a broad antifungal spectrum.

One of the major virulence factors of *C. albicans* is its ability to form a biofilm, which is a group lifestyle of microorganisms with high resistance to various antifungal agents, including fluconazole, amphotericin B, and caspofungin (Chandra et al. 2001). Our results showed that THA not only inhibited biofilm formation but also destroyed the maintenance of mature biofilms. The addition of 32 μg/ml THA almost completely inhibited biofilm formation, whereas over 70% of the mature biofilm was destroyed at this THA concentration. In addition, the transcription of EFG1 (a transcription regulator controlling filamentous growth), HWP1 and ECE1 (both mainly expressed on the hyphal surface), and ALS1 and ALS3 (two adhesin genes) was remarkably inhibited (Sundstrom 2002; Nobile et al. 2006; Araújo et al. 2017). Since hyphae growth and adhesion are important for biofilm formation, the downregulation of these genes was consistent with the inhibitory effect of THA on biofilm formation. These results indicate that THA may be a potent antifungal agent for the treatment of various forms of clinical fungal infections, such as biofilm-related infections.

Induction of intracellular ROS production in fungi is an important mechanism of action for antifungal agents, such as amphoterin B, caspofungin, and miconazole. Accumulated ROS have strong oxidant activity and can attack large molecules, such as DNA, proteins, and nucleic acids, causing irreversible damage to fungal cells (Sokol-Anderson et al. 1986; Fernández-García et al. 2022). In this study, a striking increase in the level of intracellular ROS was detected in THA-treated *C. albicans* cells. Consistent with this, a series of redox-related genes were found to be differentially expressed upon THA treatment. As a transcription factor in *C. albicans*, CAP1 can aggregate in the nucleus and regulate the transcription of many redox-related genes (Wang et al. 2006). Here, the increased expression of CAP1 in the THA-treated group might be due to the altered harmful intracellular environment, in which the fungal cells need to activate this transcription factor to upregulate the expression of specific downstream antioxidant defense-related genes; thus, an efficient oxidative stress response is initiated (Alarco and Raymond 1999; Zhang et al. 2000). Consistently, our results revealed that GRP2, GLR1, and TRR1, which are CAP1-responsive genes with antioxidant scavenging/defense properties and encode NADPH-dependent methylglyoxal reductase, glutathione reductase, and TRR1 thioredoxin reductase, respectively, were upregulated. The increased expression of these genes might be considered negative feedback in response to THA-induced ROS accumulation. SOD2 and SOD5 are two superoxide dismutases that catalyze the direct removal of ROS and play critical roles in the first line of defense against antioxidants (Tyler 1975; Luk et al. 2005). It was reported that *C. albicans* cells lacking superoxide dismutase are hypersensitive to oxidative stress (Hwang et al. 2003). Therefore, the reduced expression of SOD2 and SOD5 in fungal cells upon exposure to THA might further promote the accumulation of intracellular ROS and the corresponding oxidative damage, which leads to cell death.

Previous studies have shown that enhanced ROS production is closely related to high caspase activity (Cao et al. 2009). In the current study, caspase activity in *C. albicans* upon THA treatment was remarkably increased. The mRNA level of CaMCA1, which encodes the only known metacaspase in *C. albicans*, increased in the THA-treated groups. This result is consistent with previous reports, which showed that, as a homolog of *Saccharomyces cerevisiae* metacaspase YCA1, overexpression of CaMCA1 in *C. albicans* is closely related to ROS accumulation and caspase activation. Upon H_2_O_2_-induced oxidative damage, the *Camca1* mutant displayed higher viability and lower caspase activity as compared to the wild-type strain (Cao et al. 2009; Jung and Kim 2014). To further investigate the role of CaMCA1 in THA-induced cell death, we tested whether the presence of CaMCA1 affects the sensitivity of *C. albicans* cells to THA killing. Our results showed that, compared to the wild-type and CaMCA1-reintroduced strains, the survival rate of the *Camca1* mutant was significantly higher, accompanied by lower caspase activity. This result indicates an important role of CaMCA1 in the antifungal activity of THA.

In conclusion, this study demonstrated the activity of the natural compound THA in vitro and in vivo, which exhibited highly efficient and broad-spectrum antifungal activities. Further studies revealed that elevated intracellular ROS production and CaMCA1-mediated high caspase activity might be involved in the mechanisms underlying the antifungal action of THA.

## Data Availability

All data generated or analyzed during this study are included in this published article.
